# Trends of Cause-Specific Mortality in Union Territory of Chandigarh

**DOI:** 10.4103/0970-0218.39250

**Published:** 2008-01

**Authors:** SPS Bhatia, AK Gupta, JS Thakur, NK Goel, HM Swami

**Affiliations:** Department of Biostatistics, PGIMER, Chandigarh, India; 1 Department of Community Medicine, GMCH, Chandigarh, India

## Introduction

Data on causes of death are an important source of information on death. Such data are crucial for monitoring the reasons why people die; and targeting where, when and how health resources should be used. The reliable information on deaths compiled cause-wise is an essential input to planning, managing and evaluating the performance of the health sector in the country. The numbers of deaths by cause influence the manner in which resources are allocated to different services, programs and research activities. Also reliable information on deaths by cause is an essential input to the assessment of how cost-effective are new techniques for disease control and health promotion. The epidemiological transition, when the cause of death structure shifts profoundly from infectious to chronic diseases, is underway in most developed countries. The more developed and less developed countries differ significantly with respect to causes of death. Chronic and degenerative diseases associated with old age predominate in the West, whereas infectious and parasitic diseases associated with much younger ages prevail in less developed countries. India is undergoing rapid epidemiological transition as a consequence of economic and social change.(1) The pattern of mortality is a key indicator of the consequent health effects; but up to date, precise and reliable statistics is a big issue.

The present study was undertaken to see the trends of mortality in Chandigarh; and also a case study from a superspecialty hospital, Postgraduate Institute of Medical Education and Research (PGIMER) Hospital, was undertaken to know the causes of deaths.

## Materials and Methods

The study was conducted by collecting the death data from the District Registrar, Births and Deaths, Chandigarh; and the Postgraduate Institute of Medical Education and Research (PGIMER) Hospital, Chandigarh, for the year 2002. Deaths were classified using a standard system of ICD-10(2) (International Classification of Diseases, Tenth Revision, published by World Health Organization, Geneva). A total of 8,897 deaths were recorded in the death register for the year 2002; and out of these, 4,266 deaths were reported from the superspecialty hospital, PGIMER, only. The cause-specific mortality data were analyzed for the years 1983, 1992 and 2002 and was compared to see the trend of mortality. Prematurity is a risk factor; however, it has been taken as a cause of death in this study because secondary data was used. As a case study, further analysis of the deaths reported at PGIMER was also done for the year 2002 where the cause of death has been certified by the resident doctors of the hospital.

## Results

Causes of deaths for the years 1983,(3) 1992(4) and 2002 reported in the Union Territory (UT) of Chandigarh have been shown in Table 1. It represents all causes of mortality reported in Chandigarh, though the residence may or may not be in Chandigarh. During the last two decades, deaths due to infectious and parasitic diseases have decreased from 18.7% to 9.5% (*P* < 0.01), followed by respiratory diseases from 10.5% to 7.4% (*P* < 0.01), diseases of digestive system from 8.4% to 1.9% (*P* < 0.01) and prematurity from 6.1% to 2.8% (*P* < 0.01). However, deaths due to circulatory system diseases have increased from 18.1% to 34.9% (*P* < 0.01).

**Table 1:** Mortality trends (leading causes of death) in theUnion Territory Chandigarh during the years 1983,1992 and 2002

Cause of death	1983 N=3680	1992 N=3749	2002 N=8897
Infectious and parasitic diseases	688	462	845
	(18.7)	(12.3)	(9.5)
Circulatory system	666	1168	3086
	(18.1)	(31.2)	(34.9)
Respiratory system	388	221	659
	(10.5)	(5.9)	(7.4)
Digestive system	310	110	172
	(8.4)	(2.9)	(1.9)
Prematurity	223	76	250
	(6.1)	(2.0)	(2.8)
Nervous system and sense organs	178	84	114
	(4.8)	(2.2)	(1.3)
Genito-urinary system	169	69	83
	(4.6)	(1.8)	(.94)
Injury	362	390	1120
	(9.8)	(10.4)	(12.5)
Neoplasm	105	121	409
	(2.9)	(3.3)	(4.6)
Endocrine disorders	119	109	453
	(3.2)	(2.9)	(5.1)

Figures in parentheses are percentages

From the total deaths (4,266) reported in PGIMER, 29.3% were from Punjab, 29.1% from Chandigarh, 22.8% from Himachal Pradesh, 4.4% from Uttar Pradesh and only 3.2% from the other states of India. About 14% of deaths were at age less than 1 year; 4.2% at 1-5 years, 4% at 6-14 years; 34.2% at 15-44 years; 22.8% at 45-60 years and 20.5% at age above 60 years.

PGIMER, Chandigarh, is a superspecialty hospital, and the patients are referred from the neighboring states of the northern part of India. A total of 4,266 deaths (male: 2,719; female: 1,349) were reported during the year 2002. Table 2 shows that the most important cause of mortality was “diseases of the circulatory system” (35.7%); followed by injury, poisoning and certain other consequences of external causes (10.5%); diseases of the respiratory system (10.2%); infectious and parasitic diseases (9.7%).

**Table 2:** Comparison of indoor and emergency outdoor deaths (leading causes) in PGIMER, Chandigarh, during the year 2002

Causes of death	Total deaths N=4266	Indoor N=2719	Emergency outdoor N=1547
Diseases of the circulatory system	1524 (35.7)	795 (29.2)	729 (47.1)
Injury, poisoning and certain other	447 (10.5)	160 (5.9)	287(18.6)
consequences of external causes			
Diseases of the respiratory system	436 (10.2)	330 (12.1)	106 (6.8)
Certain infectious and parasitic diseases	412 (9.7)	330 (12.1)	82(5.2)
Certain conditions originating in the perinatal period	156 (3.7)	154 (5.7)	2(.01)
Diseases of the digestive system	109 (2.6)	72(2.6)	37(2.4)
Diseases of the genitourinary system	81 (1.9)	73 (2.7)	8 (0.50)
Diseases of the nervous system	40 (0.9)	31 (1.1)	9 (0.6)
Symptoms, signs and abnormal clinical and	541 (12.7)	297 (11.0)	244 (15.8)
laboratory findings, not elsewhere classified			

Figures in parentheses are percentages

PGIMER being a referral hospital, out of the total deaths, 63.7% were indoor deaths (patients who are admitted) and 36.3% were emergency outdoor deaths. Table 2 shows that mortality due to circulatory system diseases was more in emergency outdoor cases as compared to indoor cases (47.1% *vs.* 29.2% respectively); followed by symptoms, signs and abnormal clinical and clinical laboratory findings (15.8% *vs.* 11.0% respectively); and injury, poisoning and certain other consequences of external causes (18.6% *vs.* 5.9% respectively). But the mortality was more in the indoor patients as compared to emergency outdoor patients among the respiratory system diseases (12.1% *vs.* 6.8%) and infectious and parasitic diseases (12.1% *vs.* 5.2%).

## Discussion

The present study reveals that “circulatory system diseases” were the leading cause of death (34.9%), which was similar to the observations made by Rohina *et al.*(5) (32%) in Andhra Pradesh. The deaths due to these diseases have increased to almost double as compared to the year 1983 (18.1%), which may be due to multiple life style factors like sedentary life style, unhealthy diet, increasing tobacco and alcohol consumption, the stress of modern society and improvement in socioeconomic status. Proportionate mortality due to infectious and parasitic diseases (9.5%) was also almost similar to that reported in a study by Rohina *et al.* (12%).(5) Mortality due to infectious and parasitic diseases has decreased from 18.7% to 9.5%, followed by prematurity from 6.1% to 2.8% from the year 1983 to 2002. This may be due to the fact that Chandigarh is a planned city, and there is improvement in environmental conditions; and sanitation and better health-care facilities are available to the pregnant women.

The case study of PGIMER also reveals that from the total emergency outdoor deaths, 47.1% were due to circulatory system diseases as compared to 29.2% from the total indoor deaths. It is due to the fact that patients are referred from different parts of northern India at a very critical stage. There is a need to strengthen cardiac care services in other big hospitals in the city. The present study shows that Chandigarh is experiencing advanced epidemiological transition. There is need to create more health awareness for risk factors of circulatory diseases and standard treatment practices so that the deaths due to these diseases could be prevented. The limitations of the present study are that deaths reported from the hospitals only were medically certified and included, not including death certification of deaths which take place at home; and also this is not a community-based study.

The study shows that Chandigarh is experiencing advanced epidemiological transition, and appropriate interventions to focus on diseases of the circulatory system should be undertaken.
